# EEG microstates as biomarker for psychosis in ultra-high-risk patients

**DOI:** 10.1038/s41398-020-00963-7

**Published:** 2020-08-24

**Authors:** Renate de Bock, Amatya J. Mackintosh, Franziska Maier, Stefan Borgwardt, Anita Riecher-Rössler, Christina Andreou

**Affiliations:** 1 University Psychiatric Clinics (UPK) Basel, University of Basel, Basel, Switzerland; 2 Department of Psychology, Division of Clinical Psychology and Epidemiology, University of Basel, Basel, Switzerland; 3 Department of Psychiatry and Psychotherapy, University of Lübeck, Lübeck, Germany; 4 Faculty of Medicine, University of Basel, Basel, Switzerland

**Keywords:** Schizophrenia, Neuroscience, Biomarkers

## Abstract

Resting-state EEG microstates are brief (50–100 ms) periods, in which the spatial configuration of scalp global field power remains quasi-stable before rapidly shifting to another configuration. Changes in microstate parameters have been described in patients with psychotic disorders. These changes have also been observed in individuals with a clinical or genetic high risk, suggesting potential usefulness of EEG microstates as a biomarker for psychotic disorders. The present study aimed to investigate the potential of EEG microstates as biomarkers for psychotic disorders and future transition to psychosis in patients at ultra-high-risk (UHR). We used 19-channel clinical EEG recordings and orthogonal contrasts to compare temporal parameters of four normative microstate classes (A–D) between patients with first-episode psychosis (FEP; *n* = 29), UHR patients with (UHR-T; *n* = 20) and without (UHR-NT; *n* = 34) later transition to psychosis, and healthy controls (HC; *n* = 25). Microstate A was increased in patients (FEP & UHR-T & UHR-NT) compared to HC, suggesting an unspecific state biomarker of general psychopathology. Microstate B displayed a decrease in FEP compared to both UHR patient groups, and thus may represent a state biomarker specific to psychotic illness progression. Microstate D was significantly decreased in UHR-T compared to UHR-NT, suggesting its potential as a selective biomarker of future transition in UHR patients.

## Introduction

Psychotic disorders are complex and debilitating mental illnesses, affecting multiple domains of everyday life with potential for chronic outcomes^1^. However, timely treatment in the early stages of the illness can substantially improve clinical and functional outcomes^2,3^. Among patients with psychotic disorders, it has long been observed that a prodromal phase may precede the onset of first psychotic symptoms by several years^4^. Based on this observation, operationalized clinical criteria were developed to detect individuals at risk for psychotic disorders. The most prevalent among them are the ultra-high-risk (UHR) criteria, consisting of the presence of either (a) attenuated positive symptoms; (b) brief limited psychotic symptoms; or (c) genetic vulnerability accompanied by functional decline^5,6^. About 22–29% of UHR patients will transition to psychosis, with most transitions occurring in the first 3 years following diagnosis^7,8^. To optimize transition prediction, and thereby treatment in UHR patients, a large body of research has been devoted to identifying biomarkers that may be used to improve predictive accuracy^9–11^.

Reliable biomarkers have adequate discriminatory capacity, are present at a sufficiently early (ideally preclinical) illness stage, are selective for an illness, and are reproducible across different patient samples^12^. Moreover, to improve clinical applicability, biomarkers need to incur reasonable costs and minimal patient discomfort. One method that offers several advantages in biomarker research is resting-state electroencephalography (EEG). Apart from being inexpensive and easy to implement, resting-state EEG can capture the fast-changing dynamics of neuronal networks with high temporal resolution. Studying these networks is extremely relevant in psychosis and UHR research since multiple studies have demonstrated altered network properties in affected individuals^13–15^.

A compelling tool for studying the temporal dynamics of (eyes-closed) resting-state brain networks are EEG microstates. EEG microstates are brief (about 50–100 ms) periods in which the spatial configuration of scalp global field power remains quasi-stable before rapidly shifting to another configuration^16,17^. These spatial configurations can be clustered into a pre-defined number of configurations or classes. Four common classes, labeled A, B, C and D, explain 65–84% of EEG data variance^17^. These classes are present across different sex and age groups,^18,19^ different neuropsychiatric diseases^20^, and show cross-method consistency and high test–retest reliability^21^. Simultaneous functional magnetic resonance imaging (fMRI)-EEG studies have linked microstate classes to specific resting-state functional networks^22,23^.

Microstate classes can be characterized by a set of temporal parameters: coverage, duration, and occurrence. Previous research has identified several differences in these temporal parameters between medication-naïve patients with schizophrenia and healthy controls^24–29^. While these studies have reported changes across all microstate classes, recent meta-analyses only reported increased occurrence of microstate C and decreased duration of microstate D in patients with psychotic disorders^30,31^. Although less pronounced, some of these changes are already present in individuals with a clinical or genetic high risk for schizophrenia^32,33^, indicating that microstate alterations already occur at an early stage of psychotic disorders. The above findings suggest that EEG resting-state microstates might be a valuable candidate biomarker for the prediction of psychotic transition in UHR patients. However, no studies have yet assessed microstates in UHR patients with respect to future transition to psychosis.

The present study aimed to investigate microstate dynamics with respect to their suitability as biomarker for psychosis and transition to psychosis. To this end, we included UHR patients with and without a future psychotic transition (UHR-T and UHR-NT, respectively), first-episode-psychosis (FEP) patients and healthy controls (HC). Our comparisons were set out to examine state differences unspecific for illness progression (by comparing FEP, UHR-T, and UHR-NT to HC), state differences selective for developed psychosis (by comparing FEP to UHR-T and UHR-NT), and trait differences that reflect later transition to psychosis (UHR-T vs. UHR-NT). Based on previous studies that report microstate changes in patients with (high risk for) psychotic disorders, we expected to find both state and trait differences using EEG microstates, thereby showing the potential of EEG microstates as biomarker.

## Methods

The data presented here were collected in the context of the FePsy (*Früh****e****rkennung von Psychosen*; Early Detection of Psychoses) project, which was conducted from 2000 to 2017 with the aim to improve early detection of psychosis. The FePsy project was approved by the local ethics committee and in accordance with the Declaration of Helsinki. A detailed description of the project can be found elsewhere^34^.

### Participants

Patients were help-seeking consecutive referrals to the FePsy clinic at the psychiatric outpatient department of the University Psychiatric Clinics (UPK) Basel. Healthy controls (HC) were recruited from the same geographical area as the FEP and UHR groups. All participants gave written informed consent for participation in the project.

FEP and UHR status was determined based on the Basel Screening Instrument for Psychosis (BSIP)^35^. Participants were assigned to the FEP or UHR groups according to the respective criteria set by Yung et al.^36^. UHR participants were followed-up at regular intervals to identify those who later transitioned to psychosis (UHR-T) and those who did not (UHR-NT). The definition of transition to psychosis was made using the Brief Psychiatric Rating Scale (BPRS)^37^ according to the criteria set by Yung et al.^36^. Assessments for transition were performed monthly in the first year of follow-up, every 3 months in the 2nd and 3rd year, and yearly in the following years. The minimal follow-up duration before assigning a patient to the UHR-NT group was 3 years.

Exclusion criteria for patients were age <18 years, insufficient knowledge of German, IQ < 70, serious medical or surgical illness, previous episode of psychosis due to substance abuse, and psychotic symptomatology within a clearly diagnosed affective or borderline personality disorder. For the present analysis, we additionally excluded patients with UHR status based solely on BSIP unspecific criteria (as these criteria are associated with a lower risk of transition than the UHR criteria^38^), as well as all patients who had received antipsychotic treatment prior to the EEG recording. For healthy controls, exclusion criteria were age <18 years, current or past psychiatric disorder, family history of any psychiatric disorder, head trauma, neurological illness, serious medical or surgical illness, or substance abuse.

### EEG recording and pre-processing

Clinical EEG (20 min) was recorded by a trained lab assistant while participants were comfortably seated in a quiet room. The first 8 min of the recording, which correspond to resting-state eyes-closed EEG, were used in the present study. Every 3 min, participants were asked to briefly open their eyes for 6 s to avoid drowsiness. Additionally, participants were asked to open their eyes when behavioral (e.g., relaxation of face and neck muscles) and/or EEG signs of drowsiness (e.g., slow rolling eye movements, alpha drop-out, increased beta or theta activity) were observed. EEG was recorded with 19 gold cup electrodes (Nicolet Biomedical, Inc.), referenced to linked ears and attached according to the International 10–20 system. Impedances were always kept below 5 kΩ and sampling rate was 256 Hz.

Offline pre-processing was performed with Brain Vision Analyzer (Version 2.0, Brain Products GmbH, Munich, Germany). Raw EEG data were filtered with a bandpass (IIR; 0.5–70 Hz) and a notch (50 Hz) filter. Eyes-open epochs were removed based on marker positions and epochs with severe artefacts due to movement or poor signal were removed upon visual inspection. Channels with severe artefacts across the whole recording were interpolated. Ocular muscle artefacts were removed by means of Extended Infomax ICA. Subsequently, data were divided into 2 s segments and segments with residual artefacts were removed by means of visual inspection based on consensus between at least two independent reviewers. Finally, the data were re-referenced to the common average reference and bandpass filtered (FIR; 2–20 Hz).

### Microstate analysis

Microstate analysis was performed with the Microstate Analysis plug-in (Version 0.3; http://www.thomaskoenig.ch/Download/EEGLAB_Microstates/) for EEGLAB^39^ in Matlab 2015b. Individual microstate maps for each participant were calculated from original momentary maps using Atomize-Agglomerate Hierarchical Clustering (AAHC)^40^. The number of clusters was pre-set to four because four microstate classes have been reported to explain a large part of EEG data variance in healthy subjects^17^ and for comparability with previous studies in patients with psychotic disorders. Group model maps were calculated separately for each participant group (HC, UHR-NT, UHR-T, FEP) using a permutation algorithm that minimized common variance across subjects^26^ and class-labeled into microstates A–D by using minimal Global Map Dissimilarity and model map norms from Koenig et al.^18^. The class-labeled group model maps were then used as templates to assign individual microstate maps to the four class-labeled group maps. The microstate toolbox ignores the first and last segments and thereby only calculates non-truncated microstate parameters. The following parameters were extracted from microstate data: coverage (percentage of analysis time covered by the microstates of a given class), duration (the average duration of a microstate class in milliseconds), and occurrence/second (total number of the microstate of a given class per second).

### Statistical analysis

Group differences for each microstate parameter were investigated by means of separate 4 (microstate class) × 4 (group) repeated measures analysis of variances (ANOVAs). Since the goal of the present study was to investigate microstate dynamics with respect to their suitability as biomarker for psychosis and transition to psychosis, we carried out specific contrasts. The contrasts were planned in an orthogonal manner in that a group once split off was not brought back into a next contrast (Table 1). By comparing FEP & UHR-T & UHR-NT vs. HC (i.e., all patient groups combined compared to healthy controls), contrast I assessed changes in microstates that might reflect general illness state irrespective of diagnosis. Contrast II compared FEP vs. combined UHR-T & UHR-NT, thereby assessing state markers of established psychosis. The last contrast (contrast III) was set to examine differences that might be predictive of later transition to psychosis (UHR-T vs. UHR-NT).

**Table 1:** Orthogonal contrasts.

Contrast	Group 1		Group 2
I	FEP, UHR-T, UHR-NT	vs.	HC
II	FEP	vs.	UHR-T, UHR-NT
III	UHR-T	vs.	UHR-NT

All analyses were carried out using SPSS 25. Statistical tests in the present study are two-sided tests wherever applicable and the statistical level was set at *α* = 0.05.

#### Comparisons of class topography

A challenge in microstate analyses is that the class topographies used for the assignment of individual momentary maps and extraction of temporal characteristics may systematically differ between groups. To assess for such group differences in microstate class topographies, we used the Matlab tool Ragu (downloaded from www.thomaskoenig.ch/Ragu_src.zip^41^) to perform topographic analysis of variance (TANOVA). TANOVA uses the global field power of difference maps and non-parametric randomization statistics to quantify and assess between-group differences in scalp topography. Separate TANOVAs (5000 randomizations, L2-norm normalization of scalp field variance across sensors) were carried out for each contrast and results were Bonferroni-corrected for the total number of microstates (*n* = 4).

#### Vigilance

During eyes-closed resting-state conditions, it is possible that participants exhibit changes in arousal especially when participant groups are different clinical populations with some of them medicated. We therefore carried out an analysis assessing different stages of vigilance. We used VIGALL 2.0 (downloaded from https://research.uni-leipzig.de/vigall/) as add-in in Brain Vision Analyzer. For each participant, alpha center frequency was detected, as well as adaptations of absolute power thresholds. The different vigilance stages are: states A (1–3; alertness, relaxed wakefulness), state B (1–2/3; drowsiness), and state C (sleep) (see also Olbrich et al.^42^). Each 1-s segment was assigned to one of the states. For each state, the relative number of segments was calculated.

#### Subsidiary analyses: age mediation and moderation

As reported further below (see “Results”), between-group comparisons at baseline indicated significant differences in age between groups, with FEP being the oldest group (Table 2). As microstate parameters have been reported to variate with age^18^, we investigated whether age mediated or moderated the significant planned contrasts. The age mediation and moderation analyses were carried out using the PROCESS macro version 3.1 for SPSS^43^.

**Table 2:** Sample demographics.

	HC	UHR-NT	UHR-T	FEP			
	*n* = 25	*n* = 34	*n* = 20	*n* = 29	*F*/*χ*2	*p*	Post-hoc
Sex (M:F)	12:13	26:8	11:9	19:10	5.69	0.128	
Age (mean [SD])	22.39 (5.24)	25.32 (8.14)	25.80 (7.20)	28.68 (7.64)	3.41	0.020	FEP > HC
BPRS (mean [SD])							
Total score	–	41.62 (11.70)	41.84 (9.67)	53.81 (11.02)	10.60	<0.001	FEP > UHR-T, UHR-NT
Depression/anxiety	–	9.59 (4.32)	10.69 (2.87)	11.79 (4.44)	2.09	0.131	
Psychosis/thought disturbance	–	6.30 (2.35)	6.52 (2.12)	12.09 (3.42)	38.68	<0.001	FEP > UHR-T, UHR-NT
Negative symptoms	–	6.43 (2.51)	5.76 (2.70)	5.75 (2.81)	0.58	0.562	
Activation	–	5.80 (2.38)	5.22 (1.46)	7.21 (3.55)	3.34	0.041	FEP > UHR-T
Comorbidities (ICD-10)							
F10–F19^a^	–	1	3	1			
F30–F39^a^	–	9	16	7			
F40–F49^a^	–	3	9	0			
F60–F69^a^	–	0	0	1			
EEG total analysis time (mean [SD])	299.72 (41.16)	296.74 (48.57)	309.35 (48.14)	296.71 (45.24)	0.38	0.768	
EEG explained variance (%) (mean [SD])	76.92 (2.77)	76.75 (2.95)	75.42 (4.00)	77.25 (3.68)	1.29	0.281	

*HC* healthy controls, *UHR-NT* ultra-high-risk without transition to psychosis at follow-up, *UHR-T* ultra-high-risk with transition to psychosis at follow-up, *FEP* first-episode psychosis, *BPRS* Brief Psychiatric Rating Scale, *SD* standard deviation.

^a^F10–F19 = Mental and behavioral disorders due to psychoactive substance use; F30–F39 = Mood [affective] disorders; F40–F49 = Neurotic, stress-related, and somatoform disorders; F60–F69 = Disorders of adult personality and behavior. Significance level is 0.05; only significant differences between groups (Games-Howell corrected) are presented for post-hoc comparisons.

## Results

### Group characteristics

From a total of 162 participants with available EEG data initially recruited in the project, 53 participants were excluded ex post facto due to antipsychotic medication criteria (*n* = 18), definition of risk state based on other criteria than UHR criteria (*n* = 8), insufficient EEG quality (*n* = 24), insufficient follow-up time, or missing information on diagnosis (*n* = 3). A total of 108 participants were thus included in the analysis, consisting of 29 patients with first-episode psychosis (FEP), 20 ultra-high-risk (UHR) patients with (UHR-T) and 34 UHR patients without (UHR-NT) later transition to psychosis, and 25 healthy controls (HC). Table 2 displays group characteristics on the four study groups.

### Microstate parameters: overall results

**Fig. 1:**
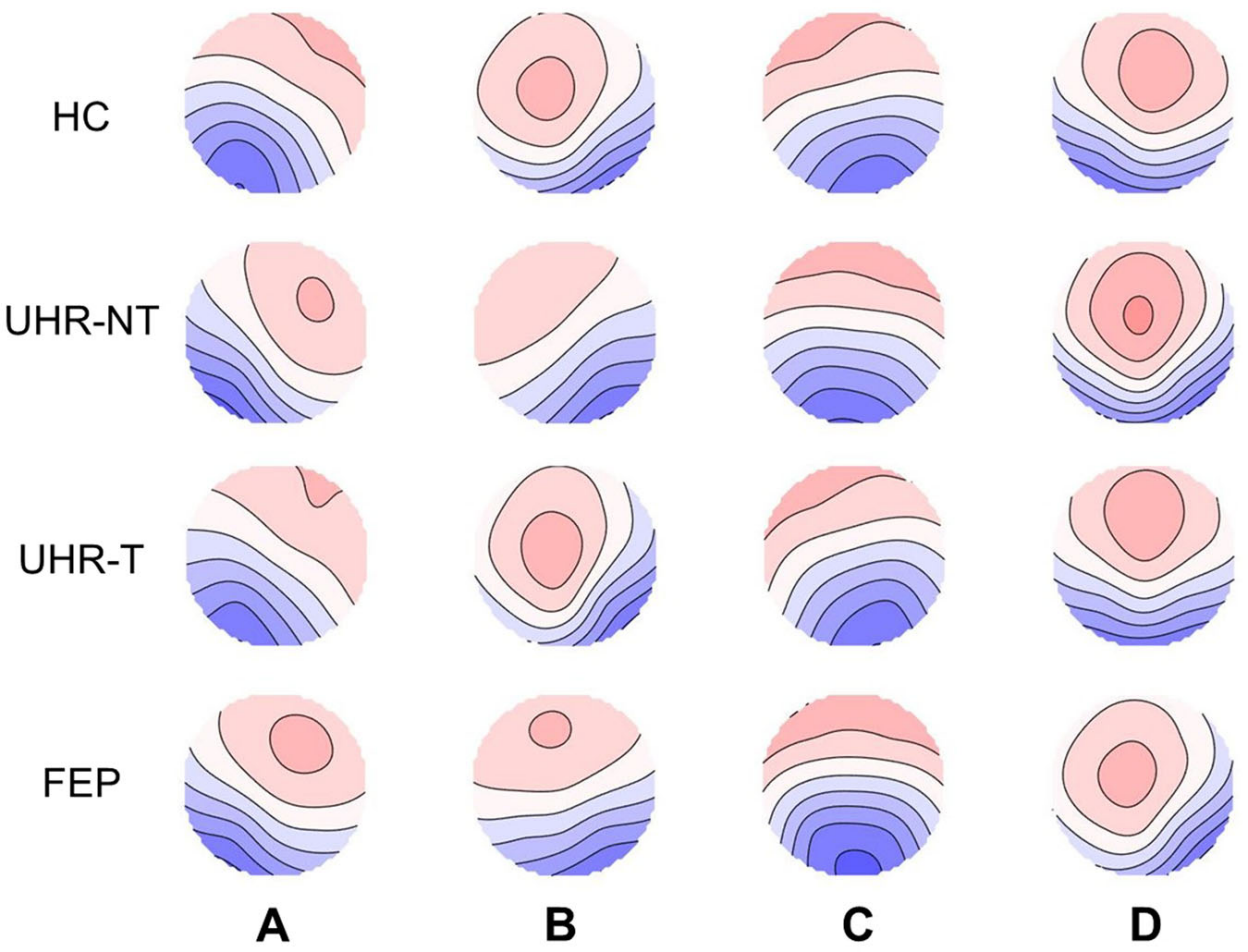
Spatial configuration of the four microstate classes. Each row displays the four topographic configurations (A-D) for each group. HC healthy controls, UHR-NT ultra-high-risk without transition, UHR-T ultra-high-risk with transition, FEP first-episode psychosis.

Class-labeled group model maps were calculated separately for each participant group and are shown in Fig. 1. The average global explained variance for all groups was 77% and did not significantly differ between groups (*F*(3, 107) = 1.293, *p* = 0.281, $\eta^2$ = 0.036) (Table 2).

### Microstate parameters: between-group differences

We found significant class x group interactions for all microstate parameters: coverage (*F*(8.291, 287.434) = 4.186, *p* < 0.001, $\eta^2$ = 0.108); duration (*F*(7.280, 252.370) = 2.130, *p* = 0.039, $\eta^2$ = 0.058); and occurrence (*F*(8.671, 300.603) = 6.334, *p* < 0.001, $\eta^2$ = 0.154). We next performed separate one-way ANOVAs to further investigate group differences in specific microstate classes. These follow-up tests revealed significant between-group differences for microstate A coverage (*F*(3, 107) = 10.582, *p* < 0.001, $\eta^2$ = 0.234), duration (*F*(3, 107) = 4.305, *p* = 0.007, $\eta^2$ = 0.110) and occurrence (*F*(3, 107) = 6.149, *p* = 0.001, $\eta^2$ = 0.151), microstate B occurrence (*F*(3, 107) = 2.756, *p* = 0.046, $\eta^2$ = 0.740), and microstate D coverage (*F*(3, 107) = 3.561, *p* = 0.017, $\eta^2$ = 0.093) and occurrence (*F*(3, 107) = 5.980, *p* = 0.001, $\eta^2$ = 0.147). There were no significant results for microstate C. Table 3 displays means for all microstate parameters.

**Table 3:** Means for all microstate parameters.

	HC	UHR-NT	UHR-T	FEP	*F*	*p*	Post-hoc
Coverage (%) (mean [SD])							
A	22.74 (5.38)	23.27 (5.08)	26.27 (6.14)	30.77 (7.50)	10.58	<0.001	FEP > UHR-NT, HC
B	20.85 (4.32)	21.46 (4.85)	21.11 (7.08)	18.04 (6.26)	2.28	0.084	
C	32.23 (6.41)	30.64 (8.64)	34.30 (5.33)	29.18 (9.28)	1.88	0.138	
D	24.18 (6.62)	24.63 (7.38)	18.32 (7.73)	22.03 (7.65)	3.56	0.017	UHR-T < UHR-NT, HC
Duration (ms) (mean [SD])							
A	66.69 (7.47)	65.49 (7.75)	70.48 (8.95)	73.71 (13.50)	4.30	0.007	FEP > UHR-NT
B	65.12 (13.98)	62.89 (8.81)	63.50 (11.10)	59.58 (11.23)	1.17	0.326	
C	77.29 (15.92)	72.94 (19.28)	76.74 (9.16)	70.84 (18.46)	0.88	0.456	
D	66.68 (15.19)	64.28 (13.00)	60.82 (14.24)	60.18 (11.55)	1.33	0.268	
Occurrence/s (mean [SD])							
A	3.47 (0.83)	3.60 (0.67)	3.79 (0.72)	4.29 (0.89)	6.15	0.001	FEP > UHR-NT, HC
B	3.31 (0.59)	3.45 (0.61)	3.34 (0.80)	2.99 (0.60)	2.76	0.046	FEP < UHR-NT
C	4.33 (0.68	4.31 (0.76)	4.60 (0.61)	4.21 (0.88)	1.08	0.361	
D	3.69 (0.64)	3.86 (0.69)	2.97 (0.76)	3.68 (0.96)	5.98	0.001	UHR-T > HC, UHR-NT, FEP

Significance level is 0.05; only significant differences between groups (Games-Howell corrected) are presented for post-hoc comparisons.

*HC* healthy controls, *UHR-NT* ultra-high-risk without transition, *UHR-T* ultra-high-risk with transition, *FEP* first-episode psychosis.

### Microstate parameters: planned contrasts

By comparing FEP & UHR-T & UHR-NT vs. HC (i.e., all patient groups combined compared to healthy controls), contrast I assessed changes in microstates that might reflect general illness state irrespective of diagnosis. We found a significant increase of microstate A coverage (*t*(104) = 2.889, *p* = 0.005, *d* = 0.580) and occurrence (*t*(104) = 2.390, *p* = 0.019, *d* = 0.514) in all patient groups compared to HC.

In order to specifically assess state markers of established psychosis, we compared FEP vs. combined UHR-T & UHR-NT (contrast II). FEP showed significantly increased microstate A coverage (*t*(104) = 4.239, *p* < 0.001, *d* = 1.006), duration (*t*(104) = 2.509, *p* = 0.014, *d* = 0.605) and occurrence (*t*(104) = 3.293, *p* = 0.001, *d* = 0.814) compared to the two UHR groups. In addition, we observed significantly decreased microstate B coverage (*t*(104) = −2.484, *p* = 0.015, *d* = −0.557) and occurrence (*t*(104) = −2.671, *p* = 0.009, *d* = −0.632) in FEP compared to UHR-T and UHR-NT combined.

The last contrast (contrast III) was set to examine differences that might be predictive of later transition to psychosis (UHR-T vs. UHR-NT). The UHR-T group showed significantly decreased microstate D coverage (*t*(104) = −3.043, *p* = 0.003, *d* = −0.840) and occurrence (*t*(104) = −4.109, *p* < 0.001, *d* = −1.244) compared to UHR-NT.

### Topographic analysis of variance (TANOVA)

#### Contrast I

The TANOVA group main effect was significant (*p* = 0.001); significant differences in topography were observed in microstates A (*p* = 0.006), B (*p* = 0.02), and D (*p* = 0.002), while the group effect for microstate C was non-significant (*p* = 0.19).

#### Contrast II

The TANOVA group main effect was significant (*p* = 0.04); differences in topography reached marginal significance in the case of microstate D (*p* = 0.05), while they were non-significant for microstates A (*p* = 0.50), B (*p* = 0.08), and C (*p* = 1.0).

#### Contrast III

The TANOVA group main effect was significant (*p* = 0.007); significant differences in topography were observed for microstates B (*p* = 0.02) and C at a trend level (*p* = 0.06), while the group effect was non-significant for microstates A (*p* = 0.14) and D (*p* = 0.11).

### Vigilance

On average, participants spent 71% in state A (awake), and 29% in state B (drowsiness). No significant interaction (group x state) (*F*(15, 624) = 0.471, *p* = 0.955) or main effect of group (*F*(3, 624) = 0.000, *p* = 1.000) was observed.

### Subsidiary analyses: age mediation and moderation

In the mediation analysis, there was no significant mediation effect of age in any of the previously significant contrasts. In moderation analyses, significant interactions between age and group were observed for microstate A contribution (*F*(1,104) = 4.132, *p* = 0.045) in contrast I (FEP & UHR-T & UHR-NT vs. HC). More specifically, this microstate parameter was increased in the patient groups FEP & UHR-T & UHR-NT compared to HC, but only in younger subjects. In addition, there were significant age x group interactions for microstate D contribution (*F*(1, 50) = 8.672, *p* = 0.005) and occurrence (*F*(1, 50) = 4.143, *p* = 0.047) in contrast III (UHR-T vs. UHR-NT), with the microstate parameters being decreased in UHR-T compared to UHR-NT only in younger subjects. The main effect of group in contrast III remained significant after controlling for age, while this was not the case for contrast I. Age did not significantly moderate contrast II (FEP vs. UHR-T & UHR-NT).

## Discussion

The aim of this study was to investigate resting-state EEG microstates as biomarker for psychotic disorders and transition to psychosis in high-risk individuals. To this end, we investigated microstate parameters in patients with first-episode psychosis (FEP), patients with ultra-high-risk for psychotic disorders (UHR), and healthy controls (HC). Moreover, we were able to directly compare EEG microstate parameters in patients with (UHR-T) and without (UHR-NT) a subsequent transition to psychosis, which makes this paper unique in EEG microstate literature.

We found increased microstate A coverage and occurrence to differentiate the three patient groups (FEP, UHR-T, and UHR-NT) from HC, as well as increased microstate A coverage, occurrence and duration to differentiate FEP from the two combined UHR groups. Previous studies have reported increased microstate A occurrence^26,27^ and coverage^26–28^ in schizophrenia patients compared to controls, but also increased microstate A coverage and duration in drug-naïve patients with panic disorders compared to healthy controls^25^ and a positive correlation of microstate A parameters with depression severity^44^. Given the high rates of non-psychotic psychiatric comorbidity in all patient groups (60%; see symptom profiles presented in Table 2), we suggest that the observed microstate A parameter increase might represent an unspecific state marker of general psychopathology.

Decreased microstate B coverage and occurrence was found to additionally differentiate FEP from the combined UHR groups. Decreased microstate B duration has been consistently reported in unmedicated schizophrenia patients compared to healthy controls^24,27,28^ (with a single exception^25^). With microstate B successfully differentiating between FEP and UHR-T & UHR-NT, who at time of the measurement were experiencing psychosis-like symptoms but had not (yet) transitioned to psychosis, these results suggest that microstate B might represent a state biomarker specific to psychotic illness progression. Interestingly, previous research by Andreou et al.^32^ found differences in the opposite direction between medicated, stable FEP and high-risk patients with respect to microstates A (decreased coverage in FEP) and B (increased coverage in FEP), suggesting a modulatory role for antipsychotic medication on these two microstates and thus providing support for their view as state markers.

The present study is the first to directly compare UHR-T to UHR-NT regarding resting-state EEG microstates at baseline. We found decreased microstate D coverage and occurrence in UHR-T compared to UHR-NT. In previous research, decreased microstate D coverage, occurrence and duration were observed in patients with 22q11 deletion syndrome, a genetic syndrome associated with high psychosis risk^33^. Microstate D coverage was also found to be decreased in unmedicated schizophrenia patients compared to healthy controls^25–27^, which was confirmed by two recent meta-analysis, including medicated and unmedicated patients with psychotic disorders^31^. Dynamics of microstate D were further suggested as candidate endophenotype by a study that compared medicated schizophrenia patients and their siblings to healthy controls and found decreased microstate D in both the patient group, as well as their siblings^30^. Our results expand upon these previous findings, indicating that decreased microstate D could be a selective trait marker that potentially predicts later transition to psychosis in UHR patients. This is in line with a previous suggestion that microstate D is associated with reality testing due to its reduction in schizophrenia^27^, hypnosis^45^, and sleep^46^. Further, microstate D was found to have shortened duration during periods of hallucinations^47^ and had increased duration at follow-up for patients that responded well to antipsychotic medication^25^. This function might be mediated by attentional processes, as microstate D has been associated with the frontoparietal attention network^48^, and suggested to be dominant during focus switching and reorientation of attention^22^, during no-task resting^49^, and involved in error-monitoring^47^.

Surprisingly, we did not find any microstate C differences in any of the studied contrasts. Microstate C was suggested to predominantly occur during activation of the salience network^22^, and would have therefore been expected to be abnormal in patients with psychotic disorders based on the aberrant salience account of psychosis^50^. On the other hand, although both aforementioned meta-analyses^30,31^ reported microstate C to be increased (coverage and occurrence) in schizophrenia patients, this effect is not very consistent across single studies, with approximately half of existing studies reporting differences in this microstate between unmedicated schizophrenia patients and healthy controls^25,27,28^, while the other half failed to observe significant differences^24,26,29^. This inconsistency may be explained by a recent observation that the optimal number of microstate maps to describe resting-state EEG data may be higher than the original four (A–D). Custo et al.^48^ have proposed a 7-map model and suggested that microstate C in the original 4-map model may, in fact, result from two spatially correlated but separate microstate topographies corresponding to different resting-state networks, which might explain the above discrepant results across studies. Further research is warranted to confirm this hypothesis, as there have not been any studies using the 7-map model in patients with psychotic disorders so far.

FEP being the oldest participant group might raise the question whether the significant age difference between HC and FEP partially accounted for our results. However, age was not a significant mediator of group differences in our subsidiary analysis. Interestingly however, differences in microstate A between HC and patient groups as well as differences in microstate D between UHR-T and UHR-NT were more pronounced in younger subjects, thus revealing age as moderator for these microstates. As Koenig et al.^18^ and Tomescu et al.^19^ demonstrated, microstate temporal parameters change throughout developmental stages from early childhood to late adulthood. This suggests that microstate differences found in this study might be influenced by altered maturation processes in patients compared to healthy controls. Indeed, recent publications have suggested that UHR patients exhibit an altered structural maturation process compared to healthy controls^51^, which was shown to be predictive of greater risk of transition to psychosis and poor functional outcomes only in younger UHR patients^52^.

To our knowledge, this is the first study investigating microstates in patients at high risk for psychotic disorders under consideration of later transition status. Its strengths include (a) the inclusion of only antipsychotic-naïve patients to ensure sample homogeneity (see Stevens et al.^53^ and Yoshimura et al.^54^ for effects of antipsychotic medication on microstate parameters), and (b) the fact that transition status for UHR patients was determined based on a sufficiently long follow-up time leaning on reported transition trajectories^34,55,56^ to minimize the amount of potential unnoticed late transitions in the UHR-NT group.

However, certain methodological points should be considered as well. First, based on previously established norms by Koenig et al.^18^, the present study assessed four microstate classes. As mentioned above, an increased number of microstates might improve the explained global variance^17^. Nevertheless, using four microstate classes has the important advantage of allowing direct comparisons of our results with previous studies in patients with psychotic disorders and high psychosis risk. Altogether with our relatively high global explained variance of 77%, we deem our current method appropriate. As a second limitation, it should be kept in mind that different pre-processing strategies, data selection methods and smoothing parameters^17^, as well as differences in microstate analysis steps (e.g., the template used for microstate class assignment^21^) may influence microstate temporal parameters. In our study, we chose pre-processing and analysis parameters such as to ensure maximum comparability with a previous study of UHR patients by our group^32^ but there may be differences compared to other studies. For future research in the field of EEG microstates, it would be very useful to harmonize methods in order to promote comparability.

## Conclusion

In sum, the present results suggest microstates A and B as state markers, respectively, for general psychopathology and psychotic symptoms, and microstate D as a trait marker that selectively identifies those UHR patients that make a future transition to psychosis. Overall, the search for robust biomarkers for transition to psychosis from the high-risk state is still a key challenge in the field of early detection research, although multiple variables have been suggested to increase predictive accuracy^57^ since the early starting points of research on prediction of psychosis transition^58^ beyond the genetic risk approach^59^. With the present study, we demonstrate the potential of EEG microstates parameters as a valuable biomarker psychosis transition in the wake of a recently published article that found microstates to successfully (accuracy 82.7%) differentiate between patients with psychotic disorders and healthy controls^60^. Further research is warranted to establish the robustness of these results in order to enhance predictive accuracy, ideally in a combined multiple variable approach.
